# The organisation and responsibility for care for older people in Denmark, Finland and Sweden: outline and comparison of care systems

**DOI:** 10.1177/14034948221137128

**Published:** 2023-01-24

**Authors:** Janne Agerholm, Jutta Pulkki, Natasja K. Jensen, Ilmo Keskimäki, Ingelise Andersen, Bo Burström, Esa Jämsen, Liina-Kaisa Tynkkynen, Pär Schön, Ann E.M. Liljas

**Affiliations:** 1 Department of Global Public Health, Karolinska Institutet, Stockholm, Sweden; 2 Faculty of Social Sciences, Tampere University, Tampere, Finland; 3 Department of Public Health, Copenhagen University, Copenhagen, Denmark; 4 Finnish Institute for Health and Welfare, Helsinki, Finland; 5 Department of Public Health, Copenhagen University, Copenhagen, Denmark; 6 Gerontology Research Center (GEREC), Tampere, Finland; 7 Faculty of Medicine and Health Technology, Tampere University, Tampere, Finland; 8 Centre of Geriatrics, Tampere University Hospital, Tampere, Finland; 9 Ageing Research Center, Stockholm Sweden

**Keywords:** Ageing, health and social care, care provision, care systems, care responsibility, older adults, health services research, health policy, health equity, Nordic countries

## Abstract

**Aim:**

To outline the organisation and responsibility for health and social care provided to older people in Denmark, Finland and Sweden.

**Methods:**

Non-quantifiable data on the care systems were collated from the literature and expert consultations. The responsibilities for primary healthcare, specialised healthcare, prevention and health promotion, rehabilitation, and social care were presented in relation to policy guidance, funding and organisation.

**Results:**

In all three countries, the state issues policy and to some extent co-funds the largely decentralised systems; in Denmark and Sweden the regions and municipalities organise the provision of care services – a system that is also about to be implemented in Finland to improve care coordination and make access more equal. Care for older citizens focuses to a large extent on enabling them to live independently in their own homes.

**Conclusions:**

**Decentralised care systems are challenged by considerable local variations, possibly jeopardising care equity. State-level decision and policy makers need to be aware of these challenges and monitor developments to prevent further health and social care disparities in the ageing population.**

## Background

Health and social care for older people in Denmark, Finland and Sweden are to a large extent tax funded and publicly organised and provided [1]. The guiding principle of the countries’ elder care policy is to make the services available to any older person in need of care, regardless of economic status and family resources [2]. The universal care systems of the Nordic countries are often considered to be very similar; however, research has shown that in recent years Finland and Sweden have become more ‘de-universalised’ compared to Denmark [1]. This paper aims to outline the organisation and responsibility for health and social care provided to older people in Denmark, Finland and Sweden. Comparing the organisation of these welfare states’ health and social care systems has the potential to identify opportunities for improvement at the system level and mutual policy learning. It will also contribute to the knowledge base of the extended research project social inequalities in ageing (sia-project.se) which this study is part of.

## Methods

The responsibility for the policy, funding and organisation of major care aspects relevant to the care of older people, including primary healthcare, specialised healthcare, prevention and health promotion, rehabilitation, and social care in Denmark, Finland and Sweden, was outlined at administrative levels (state, region, municipality). Data on the systems were collated from the literature and consultations with experts known to the research team. The findings were entered into a table, refined within the research team, and verified by the experts consulted.

## Results

The findings on how responsibilities for health and social care for older people are divided between the state, regions/hospital districts and municipalities for each country in relation to policy guidance, funding and organisation, are described below and are summarised in Table I.

**Table I.** Responsibilities for health and social care for older people in Denmark, Finland and Sweden.

	Denmark			Finland				Sweden		
	State	Region	Municipality	State	Region (from 2023)	Hospital districts	Municipality	State	Region	Municipality
**Specialised healthcare**										
Policy guidance	X			Xx	X	x	x	X		
Funding	X		X	Xx			x	X	X	
Organising		X			X	x			X	
**Primary healthcare**										
Policy guidance	X			Xx	X		x	X	X	
Funding	X		X	Xx			x	X	X	
Organising		X			X		x		X	
**Prevention and health promotion**										
Policy guidance	X		X	Xx	X		x	X		X
Funding	X		X	Xx			x	X		X
Organising			X		X		Xx		(X)	X
**Rehabilitation (medical)**										
Policy guidance	X		X	Xx	X		x	X		
Funding	X		X	Xx			x		X	
Organising		X	X		X		x		X	
**Social care for older people** ^a^										
Policy guidance	X			Xx	X		x	X		
Funding	X		X	Xx			x	X		X
Organising			X		X		x			X

a Includes home care, home nursing care, and long-term social care that also includes healthcare.

For Finland, lower case x corresponds to the current system, and capital letter X corresponds to the system introduced in January 2023.

### Denmark

In Denmark, responsibilities for healthcare are divided between the state, the five regions and the 98 municipalities. The municipalities are responsible for social care. While the state issues overall policy, assesses quality of care and the overall organisation of the healthcare system, the regions and the municipalities are responsible for the delivery of services.

The regions own and operate hospitals and allocate funding to general practitioners (responsible for primary healthcare) and medical specialists who run their own private practices. The regions do not collect taxes but receive funding from, in particular, the state and also from the municipalities based on the inhabitants’ utilisation of the regions’ treatment and rehabilitation services [3].

The municipalities are responsible for health promotion, rehabilitation and social care for older people, including home care (health and social care), home nursing and care homes. The municipalities are legally obligated to initiate preventive home visits and assist older inhabitants to live independently for as long as possible. Medically and socially vulnerable citizens aged 65–81 years receive preventive home visits according to need, whereas older people in good health receive preventive visits quinquennially until the age of 82 years, when visits become yearly [3].

### Finland

To date, the municipalities in Finland have been responsible for providing health and social care to their residents, financed through municipal and state taxes and user fees. The state steers the care system through legislation and informative guidance. For example, according to law, municipalities are obliged to assess older people’s care needs on request, and to offer care services accordingly. Long-term care is primarily provided in the older person’s home, and only medical or safety reasons form grounds for a place at a care home. Specialist care is provided by hospital districts, owned and financed by federations of participating municipalities [4].

From 2023 a new organisational structure will be implemented, and the responsibility for both health and social care will be organised by 21 new regional bodies, called wellbeing services counties. The City of Helsinki will not be part of any region but will organise health and social services within its own area. The health and social care will be financed primarily through state taxes and some user fees. The possibility for the regions to collect taxes is currently being investigated [5].

Primary healthcare, specialist care and rehabilitation, and social care (home care, i.e. health and social care, and 24-hour service housing for older people) available to all from 2023 will be organised by the wellbeing services counties. The organisation and delivery of such services are under development. Primary healthcare physicians will remain as gatekeepers for secondary healthcare, and highly specialised healthcare will be delivered by the five university hospitals funded by the government. The counties will mainly be responsible for secondary and tertiary health promotion, whereas the municipalities remain responsible for primary health promotion [5].

### Sweden

In Sweden, healthcare and rehabilitation are organised and tax funded by the 21 regions. In addition, smaller user fees for outpatient care apply to adults under 85 years. The state also provides smaller contributions to the funding of both health and social care. Through policy and funding, the state also influences health promotion and prevention, mainly delivered by the municipalities and may also involve the regions. Typically, the state decides on policy aims and directives through legislation and financial incentives. For example, care homes have to a large extent been replaced by home healthcare and social care. A care home is usually not offered until the older person’s social care needs exceed the maximum visits per day provided [6].

Except for Region Stockholm, where home healthcare is provided by the region, social care and home healthcare are organised locally by the 290 municipalities. Social care needs are assessed by the municipalities’ social care managers on request and appropriate services are offered to the older person in their home, delivered by a private or public provider of their choice. Social care is primarily tax funded in combination with patient co-payments [6].

## Discussion

In summary, the state issues policy and to some extent co-funds the largely decentralised care systems in Denmark, Finland and Sweden. Co-payment is little in Denmark and most considerable in Finland, with a larger proportion of older adults reporting higher out-of-pocket expenditures than in Sweden [7]. In Denmark and Sweden, the regions and municipalities organise the provision of care services. In Finland, the future system changes imply a transfer of the organisation of health and social care from the municipalities to the newly established regions. The primary goals of the care system changes in Finland include equal access across the country, and to provide better prerequisites for care coordination. These changes are partly driven by the rapidly ageing population, with high demand for health and social care, a major challenge that Finland shares with its Nordic neighbours [8]. Indeed, care coordination can facilitate the care to older people with complex health and social care needs [9]. Managing the responsibility for both health and social care at the same administrative level (i.e. the new regions) may facilitate care coordination. Yet, according to the experts consulted, care provision and coordination are challenged in decentralised care systems, where the provision of services is decided locally and therefore often varies considerably. Such local differences may further influence care utilisation and lead to increased inequity between different municipalities/regions [10]. Opportunities for improvement include state-level decision and policy makers to be aware of these challenges and monitor developments to prevent further health and social care disparities in the ageing populations. Further in-depth comparisons of health and social care services for older people across Nordic countries may increase policy learning and ultimately improve services.
